# Identification of clinical prognostic features of esophageal cancer based on m6A regulators

**DOI:** 10.3389/fimmu.2022.950365

**Published:** 2022-09-08

**Authors:** Huimei Wang, Yiping Zhang, Lin Chen, Yufeng Liu, Chen Xu, Dongxian Jiang, Qi Song, Haixing Wang, Liyan Wang, Yu Lin, Yuanmei Chen, Junqiang Chen, Yuanji Xu, Yingyong Hou

**Affiliations:** ^1^ Department of Pathology, Zhongshan Hospital, Fudan University, Shanghai, China; ^2^ Clinical Oncology School of Fujian Medical University, Fuzhou, China; ^3^ Department of Radiation Oncology, Clinical Oncology School of Fujian Medical University, Fujian Cancer Hospital, Fuzhou, China; ^4^ Department of Thoracic Surgery, Clinical Oncology School of Fujian Medical University, Fujian Cancer Hospital, Fuzhou, China

**Keywords:** esophageal cancer, m6A RNA methylation regulators, prognosis, signature, the cancer genome atlas, nomogram

## Abstract

**Background:**

Esophageal cancer (ESCA) is a common malignancy with high morbidity and mortality. n6-methyladenosine (m6A) regulators have been widely recognized as one of the major causes of cancer development and progression. However, for ESCA, the role of regulators is unclear. The aim of this study was to investigate the role of m6A RNA methylation regulators in the immune regulation and prognosis of ESCA.

**Methods:**

RNA-seq data were downloaded using the Cancer Genome Atlas (TCGA) database, and the expression differences of m6A RNA methylation regulators in ESCA were analyzed. Further m6A methylation regulator markers were constructed, and prognostic and predictive values were assessed using survival analysis and nomograms. Patients were divided into low-risk and high-risk groups. The signature was evaluated in terms of survival, single nucleotide polymorphism (SNP), copy number variation (CNV), tumor mutation burden (TMB), and functional enrichment analysis (TMB). The m6A expression of key genes in clinical specimens was validated using quantitative reverse transcription polymerase chain reaction (qRT-PCR).

**Results:**

In ESCA tissues, most of the 23 regulators were significantly differentially expressed. LASSO regression analysis included 7 m6A-related factors (FMR1, RBMX, IGFBP1, IGFBP2, ALKBH5, RBM15B, METTL14). In addition, this study also identified that the risk model is associated with biological functions, including base metabolism, DNA repair, and mismatch repair. In this study, a nomogram was created to predict the prognosis of ESCA patients. Bioinformatics analysis of human ESCA and normal tissues was performed using qRT-PCR. Finally. Seven genetic features were found to be associated with m6A in ESCA patients. The results of this study suggest that three different clusters of m6A modifications are involved in the immune microenvironment of ESCA, providing important clues for clinical diagnosis and treatment.

## Introduction

Esophageal cancer is a common malignancy, and in 2020, ESCA had the 7th highest incidence (604,000 new cases) and 6th highest incidence (544,000 deaths) of all cancers (1). ESCA has a high mortality rate due to the lack of effective diagnostic and treatment strategies.

N6-methyladenosine (m6A) is the most common epigenetic RNA modification that plays an important role in the regulation of malignancies (2). Despite the potential of m6A for the diagnosis and treatment of ESCA (3, 4), its potential targets and mechanisms remain unclear.

m6A refers to the methylation reaction at the sixth position of adenosine. m6A methylation abnormalities play an important role in many diseases, especially in tumors (5). Methyltransferases (METTL3, METTL14, METTL16, WTAP, KIAA1429, ZC3H13, RBM15), demethylases (FTO, ALKBH5), binding proteins (HNRNPC, HNRNPA2B1, YTHDF1, YTHDF2, YTHDC2, YTHDC2, YTHDC1). m6A methylation regulators have important effects on ESCA progression, proliferation, migration, and invasion. hNRNPA2B1 affects the prognosis of ESCA by regulating the miR-17-92 cluster as an oncogenic factor (6). In addition, HNRNPA2B1 promotes ESCA progression through upregulation of fatty acid synthase ACLY, ACC1 (7). ALKBH5 exerts tumor suppressive effects by inhibiting miR-194-2 biogenesis through demethylation of pri-miR-194-2, thereby inhibiting RAl1 (8). Another study showed that FTO is involved in oncogenesis of ESCA through upregulation of MMP13 (9). In addition, METTL3 may also promote ESCA proliferation and invasion by regulating multiple pathways, such as AKT (10), Notch (11), COL12A1/MAPK (12) and other signaling pathways.

In addition, Xu et al. (13) demonstrated that eight regulators (KIAA1429, HNRNPC, RBM15, METTL3, WTAP, YTHDF1, YTHDC1, YTHDF2) were significantly upregulated in ESCA tissues. The above results suggest that the prognostic features of two genes, ALKBH5 and HNRNPC, have a predictive effect on prognosis. In another study (7), HNRNPA2B1, ALKBH5 was the prognostic signature consisting of HNRNPA2B1 and ALKBH5 (7). In addition, a recent study found that m6A methylation regulators may be important mediators of PD-L1 expression and immune cell infiltration, which may strongly influence the tumor microenvironment of esophageal squamous cell carcinoma (ESCC) (14). Furthermore, Saiyan et al. (15) suggested that Flap endonuclease 1 Facilitated hepatocellular carcinoma progression by enhancing USP7/MDM2-mediated P53 inactivation. However, previous bioinformatics studies were relatively simple: no multi-omics integration analysis or assessment of tumor mutation burden (TMB) was used, or focusing on exploration of the single gene, or without experimental validation. The aim of this study was to investigate the molecular targets and therapeutic mechanisms of ESCA.

## Methods

### Data acquisition and processing

RNA transcriptome data (FPKM) format was obtained from the TCGA public database (https://portal.gdc.cancer.gov/), along with copy number variation (CNV), somatic mutations, corresponding clinical data, TNM classification, survival information, and prognostic data for ESCA patients. To ensure data consistency, FPKM was transformed into transcripts per kilobase (TPM) values. Patients with no clinical information were excluded from this study. Finally, 161 ESCA samples and 11 adjacent tissues were included in the analysis. For CNV analysis, the "Circos" R package was used, and CNV genes were mapped on 23 chromosome pairs. Somatic mutation data were obtained from the TCGA database, and somatic mutation data were visualized using the "maftools" software (16). In addition, a TMB examination was performed for each patient.

### Construction and prediction of predictive features

First, the differences in m6A-related regulators expression between ESCA samples and normal tissues, and the relationship between regulator expression and prognosis of ESCA patients were analyzed. To determine the prognostic value of m6A-associated regulators, TCGA-ESCARNA-seq candidate risk regulators were selected in combination with LASSO regression analysis to reduce the dimensionality and select representative indicators. Subsequently, the selected genes were subjected to dimensionality reduction analysis to determine whether the selected genes had independent prognostic value. Finally, the minimum criterion was used to determine the corresponding specification coefficients. The regression coefficients were estimated based on the LASSO regression model, and the results were calculated as follows.


$$\text{riskScore}=\sum_{i}\text{Coefficient}\left({\text{hub gene}}_{\text{i}}\right)\times \text{mRNA Expression}\left({\text{hub gene}}_{\text{i}}\right)$$

ESCA patients were divided into low-risk and high-risk subgroups according to median risk score.

### Genomic and functional analysis

Gene ontology (GO) functional analysis is a common method for large-scale functional enrichment studies, including biological process (BP), molecular function (MF), and cellular component (CC). GO analysis was performed using the clusterProfiler (17) R package, based on differential gene expression analysis between high-risk and low-risk groups. False discovery rate q<0.05 was considered statistically significant.

Gene set enrichment analysis (GSEA) is a computational method to analyze whether statistical differences exist in a particular gene set. In this study, the GSEA method was used to analyze TCGA-ESCA RNA-seq data to explore the differences in BP between different sets." The h.all.v7.2.symbols.gm t set was downloaded from the MSigDB (18) database and used for GSEA. p<0.05, statistically significant.

### Quantitative validation of pivotal genes using reverse transcription polymerase chain reaction

The expression of IGFBP2, ALKBH5, FMR1, RBMX in 15 pairs of ESCA and adjacent esophageal tissues was detected by quantitative reverse transcription polymerase chain reaction (qRT-PCR). IGFBP2, ALKBH5, FMR1, RBMX, were acquired from BioSune (Shanghai, China). qRT-PCR analysis was performed using the Hieff® qPCR ® qRT-PCR system (Applied Biosciences, USA, USA) was used for qRT-PCR analysis. Reactions were performed at 95°C for 10 min,95°C, 40 cycles for 15 s, and 60°C for 1 min. The relative levels of gene expression were calculated by the 2-Δ Ct method using GAPDH as a reference gene. The study was conducted according to the Declaration of Helsinki of the World Medical Association. The School of Clinical Oncology of Fujian Medical University approved the use of human tissues.

### Consistent clustering study of m6A-related genes

To assess genetic identity, molecules significantly associated in the m6A risk model were analyzed using the Spearman method. p values were adjusted with the Benjamin-Hochberg test. When the absolute correlation coefficient was greater than 0.3 and P<0.01, it was significantly associated with genetic correlation. Tumor samples were clustered into distinct GeneClusters using the Kaplan-Meier method, which partitions around significantly correlated molecular expression and Euclidean measurement distances. Specifically, clustering analysis was performed using the ConsensusClusterPlus (19) R package, with 1000 cycles of calculation to ensure stability and reliability of the classification. To study the signature genomes, the Boruta algorithm was used to perform a dimensionality reduction analysis of significantly related genomes. Then, two classes, signature gene A and signature B, were clustered according to signature gene expression changes and visualized using the ComplexHeatmap package in the R software.

### Copy number variation analysis

For CNV analysis, masked copy number segment datasets for different risk groups were downloaded from TCGA-ESCA. The data were examined using GISTIC 2.0 (20). GenePattern 5 was used for the above analysis.

### Construct and validate the prediction nomogram

To improve the value of the signature in clinical practice, clinical factors (T, N, M, TNM) and m6A risk score were used as prognostic nomograms to evaluate the probability of OS occurrence in ESCA patients at 1, 2, and 3 years. To quantify the discriminatory performance of the nomogram, the concordance index (C-index) of T, N, M, m6A risk score, TNM, TNM+m6A risk score were compared. Calibration curves, time-dependent receiver operating characteristic (ROC) curves, and decision curve analysis (DCA) were used to examine the TNM and TNM+m6A risk scores.

## Results

### Genetic variation of m6A-related genes

**Figure 1:**
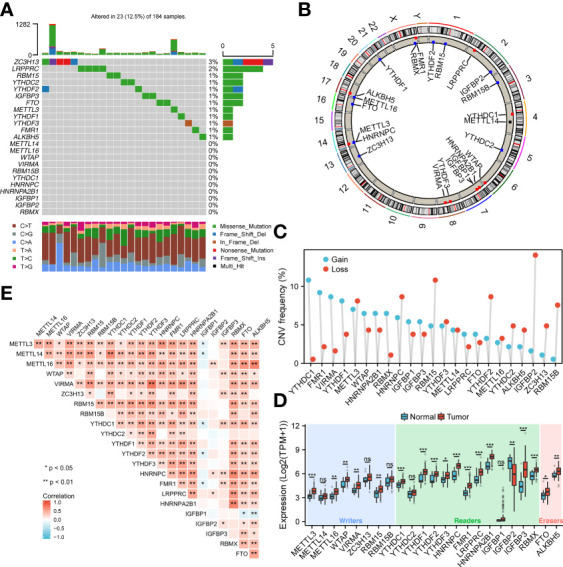
Genetic variants of m6A-related genes. **(A)** SNP of m6A related genes in 23 samples. **(B)** Location of CNV alterations on chromosomes. **(C)** Frequency of CNV in m6A related genes. Blue represents amplification, orange represents deletion. **(D)** Genetic variants of m6A-related genes. **(E)** Diagram showing the relationship between different m6A-related genes through Pearson correlation analysis. Red and blue represent a positive and negative correlation, respectively. *p < 0.05, **p < 0.01, ***p < 0.001; ns, not significant.

To analyze the overall expression of m6A-related genes in ESCA patients, this paper first analyzed mutations and gene expression levels, including single nucleotide polymorphisms (SNPs), CNV, and gene expression. Among the 172 samples, 23 samples showed SNPs of m6A-related regulators, mainly missense mutations (**Figure 1A**). Subsequently, we summarized the incidence of CNV for the 23 m6A-associated regulators in the ESCA samples. **Figure 1B** shows the altered CNV chromosomal location. CNV alterations were widespread in m6A-related genes, with reduced copy number in most patients (**Figure 1C**). In addition, the mRNA expression levels of m6A-related genes were analyzed between ESCA samples in this study, and the results showed that all genes were differentially expressed except METTL14, ZC3H13, RBM15, YTHDC2, and IGFBP1 (**Figure 1D**). Spearman correlation analysis was applied to correlate the 23 m6A RNA methylation regulators. From **Figure 1E**, the transcriptome associations were explored, and we suggested that there are close correlations among writers, erasers and readers. The correlation between RBMX and HNRNPC, HNRNPA2B1 was the highest (P<0.01), while the correlation between YTHDF3, VIRMA was the highest (P<0.01) (P<0.01).

### Construction and validation of a prognostic risk model based on seven m6A methylation regulators

**Figure 2:**
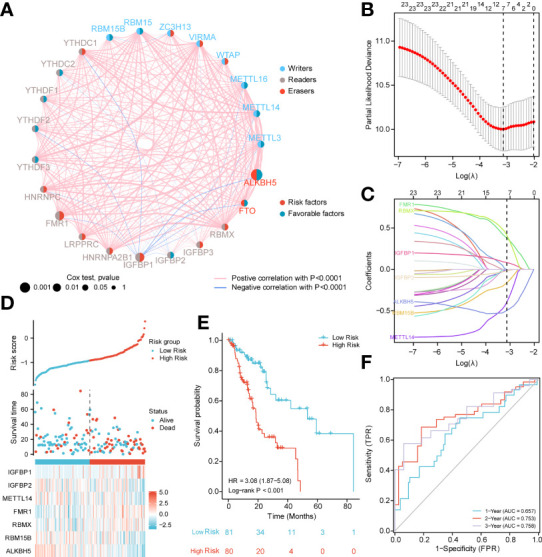
PPI network and prognostic signatures construction and prediction. **(A)** Interaction among m6A-related regulators. Circle size indicates the effect of each gene on survival, the larger the size, the greater the effect; on the right half of the circle, red represents risk prognostic factors and blue represents favorable factors; on the left side of the circle, red represents recognition proteins (readers), blue represents methyltransferases (writers), and brown demethyltransferases (erasers); lines that connect genes exhibit genetic interactions, red and blue represent positive and negative associations, respectively. **(B)** Partial likelihood deviance of different numbers of variables. One-thousand-fold cross-validation was applied for tuning penalty parameter selection. **(C)** LASSO analysis identified seven m6A-related genes in the 23 m6A-related regulators cohort. Each curve corresponds to one gene (cyan, FMR1; green, RBMX; pink, IGFBP1; brown IGFBP2; blue, ALKBH5; orange, RBM15B; purple, METTL14). **(D)** Risk score, distribution of patient survival status between the low- and high−risk groups, and expression heat maps of seven m6A-related regulators. **(E)** Kaplan–Meier curves indicated that there is a strong relationship between high and low m6Ascore and the overall survival rate. **(F)** ROC curve was applied to assess the predictive efficiency of the prognostic risk signature.

Next, we analyzed the role of m6A-related regulators in ESCA patients. The m6A regulatory network shown in **Figure 2A** reveals the interactions with m6A-related genes, nodes, and their role in ESCA prognosis. It was found that not only the m6A regulators share the same functional class, but also the expression of functional class m6A regulators was significantly correlated.

In addition, to quantitatively evaluate the effect of m6A-related regulators on the prognosis of each ESCA patient, we constructed a risk model of m6A-related regulator expression. First, based on the results of LASSO regression analysis, the minimum-minimum criterion and optimal-minimum criterion M6A-related genes, including FMR1, RBMX, IGFBP1, IGFBP2, ALKBH5, RBM15B, METTL14, were used (**Figure 2B–C**). Meanwhile, the penalty coefficients of the characteristic regulators were calculated using LASSO analysis, and the risk index was established by multiplying the gene expression by the corresponding coefficients. The risk score of each sample was then calculated based on the median of m6A scores and divided into two groups of low risk and high risk. The risk score distribution, survival status and characteristic gene expression patterns are shown in **Figure 2D**. Kaplan-Meier survival analysis showed that OS was significantly lower in the high-risk group ESCA patients than in the low-risk group (log-rank p<0.001, **Figure 2E**).

The sensitivity and predictive specificity of the risk scores were investigated using ROC curve analysis. The AUC values were 0.657, 0.753, 0.758, and 0.758, respectively (**Figure 2F**). The AUC values showed that the risk scores significantly predicted the prognosis of patients with ESCA.

### GO enrichment and genome enrichment analysis

**Figure 3:**
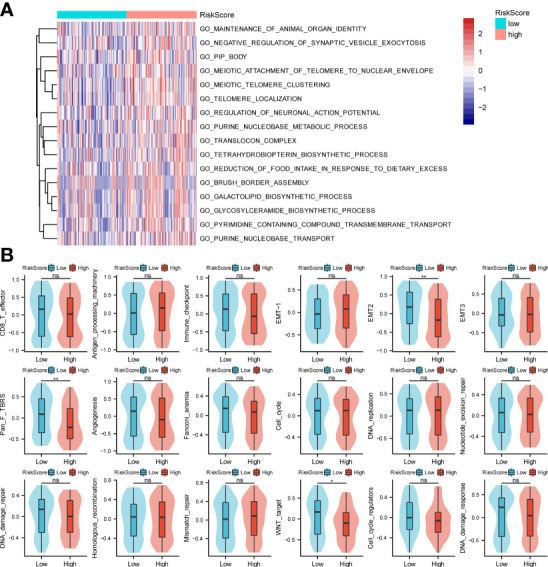
GO enrichments and gene set variation analysis. **(A, B)** Bioinformatics analysis of low- and high-risk groups. *p < 0.05,**p < 0.01, ns, not significant.

In this study, GO analysis was used to explore the biological functions of the low-risk and high-risk groups. The results showed the processes of organ identity maintenance, negative regulation of synaptic vesicle extravasation, tubular boys, meiotic telomere and nuclear envelope attachment, meiotic telomere aggregation, telomere localization, neuronal action potential regulation, purine nucleotide metabolism in the high-risk group animals. decoder complex, tetrahydrobiopterin biosynthesis process, reduced food intake, brush border assembly, galactolipid biosynthesis, glycosylceramide biosynthesis, transmembrane transport of pyrimidine compounds, and purine nucleobase transport (**Figure 3A**). Next, we performed the GSEA analysis shown in **Figure 3B**. The high-risk group was associated with antigen processing mechanisms, EMT-1, and mismatch repair. The low-risk group was correlated with CD8-T, immune checkpoint, EMT2, pan-F TBRS, angiogenesis, Fanconi anemia, DNA damage repair, WNT target, and DNA damage response.

### Validation of *IGFBP2 ALKBH5, FMR1*, and *RBMX* by qRT-PCR

**Figure 4:**
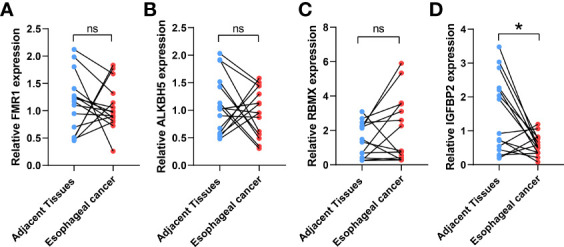
Validation of the expression levels of target *IGFBP2*. The *IGFBP2* expression in ESCA (n = 15), and adjacent normal tissues (n = 15) was evaluated by qRT-PCR including FMR1 **(A)**, AKBH5 **(B)**, RBMX **(C)**, IGFBP2 **(D)**; the results were analyzed using paired sample t test. Results are expressed as mean ± standard deviation (SD). *p< 0.05, *p< 0.05, ns, not significant.

IGFBP2, ALKBH5, FMR1, and RBMX were selected as the study subjects and validated by qRT-PCR method (**Table 1**). RESULTS: The expression level of IGFBP2 in normal tissues was significantly higher than that in ESCA tissues (**Figure 4**).

**Table:** Table 1 Primers of IGFBP2, ALKBH5, FMR1, RBMX, and GAPDH.

Primer	Forward (5′ to 3′)	Reverse (5′ to 3′)
*IGFBP2* *ALKBH5* *FMR1* *RBMX*	TGCAGACAATGGCGATGACC GACAAGGAAGAGAACCGGCG GCCAAAGAGGCGGCACATAA CCCAGCAGACGCTAAGGATG	GGTGCTGCTCAGTGACCTTC GCATCTTCACCTTTCGGGCA CGCAGACTCCGAAAGTGCAT CTACGAGAGGGCAGCGGTTC
*GAPDH*	GGAGCGAGATCCCTCCAAAAT	GGCTGTTGTCATACTTCTCATGG

### Constructing genetic traits based on the m6A risk model

**Figure 5:**
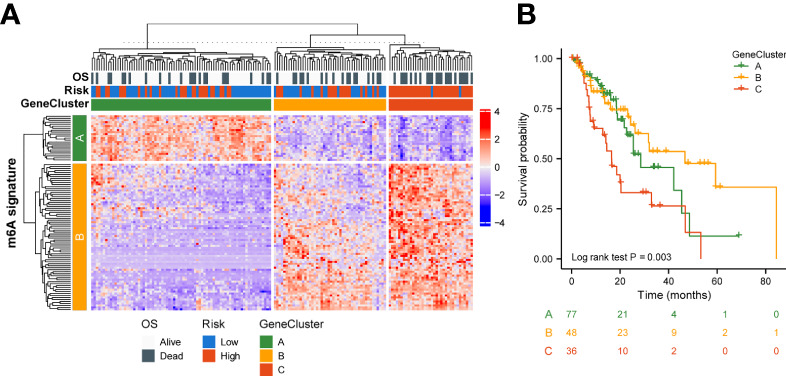
Genetic signature of risk grouping. **(A)** Relationship between GeneCluster groups, m6A signature groups, m6A risk scores, and clinical prognosis. **(B)** Kaplan–Meier curves indicate that there is a strong relationship between GeneClusters A, B, and C and the overall survival rate.

To better understand the biology of phenotypes associated with the m6A risk model, genes significantly associated with m6A risk scores were analyzed using the Spearman method (Cor|>0.3 & p.adjusted<0.01). A total of 741 associated genes were identified. Subsequently, based on the expression of these genes, the unsupervised clustering method was used to classify ESCA patients into three subtypes, named GeneClusters A, B, and C. The dimensionality of the associated gene clusters was reduced using the Boruta algorithm to obtain the signature gene clusters. Based on the trend of signature gene expression, the signature genes were classified into two groups, A and B. The relationship between GeneCluster groups, m6A signature group, m6A risk score and clinical prognosis was further analyzed (**Figure 5A**). Meanwhile, the results of survival analysis showed significant differences in the prognosis of patients with the three gene subtypes, with the GeneCluster C group having the worst prognosis (log-rank p=0.003, **Figure 5B**).

### Effect of genetic variant risk score

**Figure 6:**
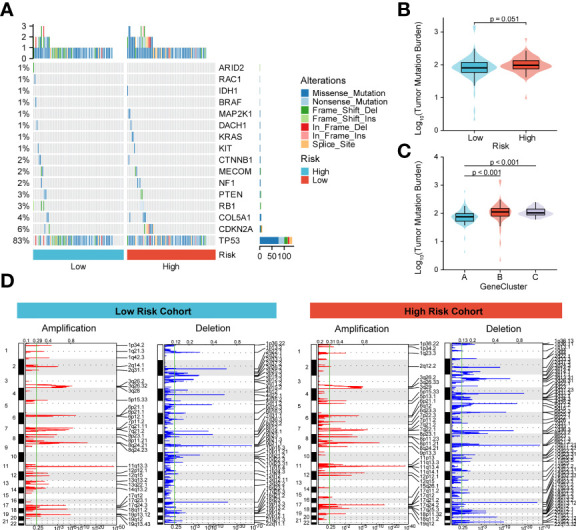
Molecular profiling of high and low m6A score groups. **(A)** Distribution of driving genes during common tumorigenesis between high and low m6A score samples; **(B, C)** Tumor mutation burden distribution in the different m6A score samples and GeneCluster groups; **(D)** Distribution of copy number amplifications and deletions in high and low m6A score samples.

To better understand the effect of high and low m6A scoring methods on the level of genetic variants, this study analyzed single nucleotide mutations in driver genes during common tumorigenesis, with differences in SNP levels between groups (**Figure 6A**). The overall level analysis showed that TMB (P=0.051, **Figure 6B**) was slightly correlated between the low-risk and high-risk groups, and TMB was significantly lower in GeneCluster A than in groups B and C (**Figure 6C**). In addition, the study of CNV variation frequency showed that the variation of gene CNV in the high-risk group was mainly focused on gene amplification (**Figure 6D**), while the low-risk group deleted gene copy number relatively (**Figure 6D**).

### Construction of a clinical prediction model based on the m6A risk score

**Figure 7:**
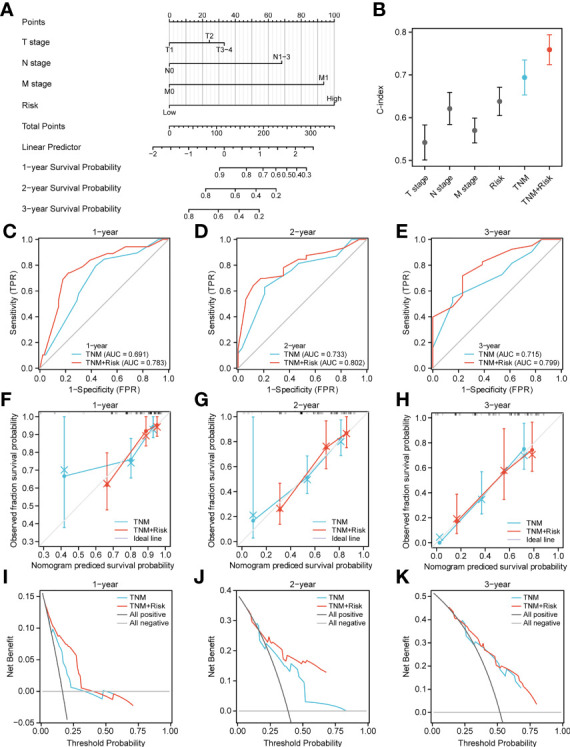
Nomogram analysis. **(A)** Nomogram composed of stage, T, N, M, and risk score for the prediction of 1-, 2-, and 3-years OS probability. **(B)** C-index analyses of T stage, N stage, M stage, risk score, TNM, TNM +risk score. **(C–E)** ROC curve for the nomogram based on TNM prognosis model and TNM +risk score model 1-, 3-, and 5-y survival. **(F–H)** Calibration plot of the TNM prognosis model and TNM +risk score model for 1-year **(F)**, 2-year **(G)**, and 3-year **(H)** OS. **(I–K)** DCA curve of TNM prognosis model and TNM +risk score for 1, 2, and 3 years.

Next, to quantify OS prediction, we combined risk scores with independent clinical characteristics (T, N, M, and TNM) to construct a nomogram (**Figure 7A**). To verify the different predictive effects of m6A risk scores on the combined T, N, and M stages, this study compared the T, N, M,m6A risk scores using the scales of the TNM risk scale; and a TNM+m6A risk score model was developed (**Figure 7B**). The results showed that the TNM+m6A prediction model had a better predictive effect. To verify the prognosis of TNM and the predictive value of the TNM+m6A risk score model, time-dependent ROC curves were established (**Figures 7C–E**). the AUC of the TNM model for 1, 2, and 3-year OS were 0.691,0.733, and 0.715, respectively; the AUC of the TNM+m6A risk score model for OS 1,2, and 3-year OS were 0.783, These results suggest that the TNM+ risk score model has a higher predictive value than the TNM model. Also, calibration curves were generated to test the correctness of the models. With the calibration curves, we found that the survival curves predicted by both models at 1, 2, and 3 years were very close to the observed survival curves, indicating that the nomograms were highly predictive (**Figures 7F–H**). In addition, DCA showed that the TNM+m6A risk score model had a broader clinical benefit than the TNM prognostic model, but the benefit of 3-year OS was similar (**Figures 7I–K**).

## Discussion

ESCA is a lethal malignancy. Despite advances in surgery, radiation therapy, chemotherapy and immunotherapy, the 5-year survival rate of ESCA remains low due to late detection and lack of precise treatment (21). Therefore, it is important to gain insight into the mechanisms of oncogenicity of ESCA. ESCA is closely associated with lifestyle environmental factors that can alter genomic inheritance and epigenetics (22). m6A modification is a novel regulatory mechanism of eukaryotic gene expression that controls gene expression through reversible epigenetic modifications (23).

In the present study, CNV alterations in the m6A regulator were prevalent in ESCA patients and mostly concentrated in copy number deletions. However, SNP was low in the m6A-regulator. We further demonstrated general differences and positive correlations in the expression of the 23 m6A-gene regulators in ESCA. Next, prognostic scores (high vs. low risk) were established based on the expression levels of FMR1, RBMX, IGFBP1, IGFBP2, ALKBH5, RBM15B, and METTL14 genes. These patterns could well predict the survival of ESCA patients. Initial analysis of biological functions in the high-risk versus low-risk groups was performed by GO functional annotation and genomic variation analysis. Based on this, the TCGA-ESCA cohort was clustered according to the high-risk score moderator. There were significant survival status differences among the three GeneClusters. In addition, there were some differences between the high-risk and low-risk groups according to prognostic characteristics, with CNV alterations mainly focused on gene amplification and the opposite in the low-risk group. Finally, a nomogram was constructed combining risk scores and independent clinical characteristics (T, N, M and TNM). The results showed that the TNM+m6A model was the best predictor. The calibration curves showed a significant agreement between prediction and actual survival probability.

First, 17 m6A regulators (METTL3, METTL16, WTAP, VIRMA, RBM15, YTHDC1, YTHDF2, YTHDF3, HNRNPC, FMR1, LRPPRC, HNRNPA2B1, IGFBP2, IGFBP3, RBMX, FTO, ALKBH5 gene expression differences. The expression of IGFBP2 in normal tissues was significantly higher than that in ESCA tissues as confirmed by qRT-PCR; IGFBP2 is involved in various oncogenic processes, such as epithelial-to-mesenchymal transition, cell migration, invasion, angiogenesis, stemness, transcriptional activation, epigenetic programming, etc. A recent study showed that rs1470579 CC genotype IGFBP2 is protective against adenocarcinoma of the esophagogastric junction (24). Based on previous findings, we suggest that IGFBP2 may be a key regulator affecting the prognosis of patients with ESCA. reduced IGFBP2 gene expression may be associated with poor prognosis. Genomic mutation analysis showed that SNPs and CNVs of gene regulatory genes were associated with ESCA.

LASSO regression analysis showed that the prognostic features of ESCA patients-FMR1, RBMX, IGFBP1, IGFBP2, ALKBH5, RBM15B, METTL14-predicted the OS of ESCA patients. ALKBH5 promotes ESCC proliferation and its mechanism of action is cell cycle regulation (25). IGFBP1 has a regulatory role in cell proliferation and invasion under the regulation of miRNAs. miRNAs regulate oncogenes with regulatory cell proliferation and invasive effects. For example, miR-454-3P can act on IGFBP1 through ERK and AKT signaling, thereby inhibiting its proliferation, invasion, and apoptosis (26). However, the mechanism of action of FMR1, IGFBP1, RBM15B, and METTL14 is still unclear. We will explore their relationship with the development of ESCA in a future study. The predictive potential of these seven factors combined is much greater than that of individual factors. Although the ROC curve did not show strong predictive power in 4-5 years, the number of patients in year 4-5 was too small, which may lead to an unstable ROC curve.

In addition, this study also analyzed the GO enrichment and GSVA of ESCA patients based on risk scores using TCGA information. The high-risk group may be involved in telomere localization, base metabolism, base translocation, and mismatch repair. This may be the reason for the poor prognosis of ESCA due to risk modifiers.

On this basis, the TCGA-ESCA cohort was clustered according to the high-risk score moderator. The results of the survival analysis showed significant differences in prognosis among the three gene cluster groups, with the GeneCluster C group having the worst prognosis. Most samples had high risk scores in GeneCluster C. These results suggest that the regulators involved in the m6A risk model are significantly correlated with ESCA characteristics.

We then combined risk scores and independent clinical characteristics (T, N, M, TNM) to construct a nomogram that allowed the prediction to be quantified. The results showed that the TNM combined with the risk score model was able to predict the prognosis of ESCA patients. This improves the value of this prognostic feature for clinical application.

The study has several limitations. First, the lack of complete clinical data from TCGA may affect the results; therefore, the statistical power may not be high. Further improvement of sample size, sequencing data and clinical information is essential. Second, the mechanism of IGFBP2's effect on the prognosis of ESCA has not been explored in depth. Therefore, this study will focus on the mechanism of IGFBP2's action as a tumor suppressor. Finally, this study was based on bioinformatics and qRT-PCR techniques to analyze the results of ESCA patient tissues.

## Conclusions

The above results suggest that abnormal expression of 17 m6A- RNA methylation regulators is associated with survival outcome in ESCA patients. The risk score was then combined with TNM to quantify OS prediction. This study highlights the important role of RNA modifications in the formation of ESCA and also provides potential biomarkers for the selection of therapeutic approaches.

## Data availability statement

The studies involving human participants were reviewed and approved by the Oncology Ethics Committee of Fujian Province, China (No. K202-027-01). The patients/participants provided their written informed consent to participate in this study.

## Ethics statement

The studies involving human participants were reviewed and approved by Effect of SASS6 gene on proliferation of esophageal carcinoma cells and its molecular mechanism (No.K202-027-01). Ethics Committee: Oncology Ethics Committee of Fujian Province, China. The patients/participants provided their written informed consent to participate in this study.

## Author contributions

YX and YH proposed and managed the study. HMW and YZ wrote the manuscript. LC and YFL collected the material and CX, DJ, QS, and HXW performed the bioinformatic analysis. LW and JC provided the samples. Data analysis consisted of YL and YC. All authors contributed to the article and approved the submitted version.

## Funding

Shanghai Municipal Health Commission (#20214Y0275), National Natural Science Foundation of China (81702372), Shanghai Science and Technology Commission (19441904000), Shanghai Key Clinical Specialties (#shslczdzk01302), Shanghai Science and Technology Development Fund (#19MC1911000), National Natural Science Foundation of China ( U21A20377), Natural Science Foundation of Fujian Province (#2020J011123).

## Acknowledgments

Thanks to editage for providing thesis revision support.

## Conflict of interest

The authors declare that the research was conducted in the absence of any commercial or financial relationships that could be construed as a potential conflict of interest.

## Publisher’s note

All claims expressed in this article are solely those of the authors and do not necessarily represent those of their affiliated organizations, or those of the publisher, the editors and the reviewers. Any product that may be evaluated in this article, or claim that may be made by its manufacturer, is not guaranteed or endorsed by the publisher.
